# Do we need empirical research on the use of trolley dilemmas in applied ethics? Reply to commentary by Heidi Matisonn

**DOI:** 10.1177/1556264620939805

**Published:** 2020-07-08

**Authors:** Gry Oftedal, Ingrid H. Ravn, Fredrik A. Dahl

**Affiliations:** 1 Department of Philosophy, Classics, History of Art and Ideas, University of Oslo, Oslo, Norway; 2 Department of Nursing and Health Promotion, Oslo Metropolitan University, Oslo, Norway; 3 Health Services Research Unit, Akershus University Hospital, Lørenskog, Norway

**Keywords:** ethical judgment, vaccine, trolley dilemmas, deontological positions, immediacy

In Heidi Matisonn’s commentary (Matisonn, 2020), she questions the rationale behind our research on the possibility of a correlation between how research ethics committee members and specialist nurses respond to trolley dilemmas and how they respond to various vaccine research scenarios (Dahl & Oftedal, 2019; Oftedal et al., 2020). Since we acknowledge that trolley problems were not originally meant to be used in this way, she finds it curious that we would take the time to investigate our research questions at all. The main reason is that she finds our conclusion to be obvious from the outset. The vaccine scenarios are just too different from the trolley dilemmas to expect any correlation between responses.

In this response, we argue that our conclusion is not that obvious and that it can be of interest to compare how people respond to trolley dilemmas with how they respond to more complex ethics problems.

One significant rationale for our research is how vaccine scenarios are compared to and aligned with trolley dilemmas in the existing literature. Although trolley problems were not designed to tell us about people’s moral intuitions in different contexts, trolley problems are frequently used this way in the literature. Matisonn claims that we do not support this assertion with references, which is simply not correct. As referenced in our articles, we find in the medical ethics literature that trolley problems are used to guide reasoning about vaccine trials, sham surgery, and so-called “challenge studies” (Albin, 2005; Andrade, 2019; FitzPatrick, 2003; Fritz, 2015; Hope & McMillan, 2004; Rosenbaum, 2018; Spier, 2011). Further, in several contributions to the field, vaccination contexts are interpreted to mirror some key aspects of trolley problems (Bartels, 2008; Bialek & De Neys, 2016; Wiss et al., 2015; Young & Koenigs, 2007). Taking this literature into account, our study contributes directly to on-going discussions in the field. We show empirically that it is problematic to use trolley problem reasoning in this way, and our study thus serves as an empirical basis from which to criticize certain actual uses of trolley problems.

We agree with Matisonn’s analysis of differences between vaccine problems and trolley dilemmas and that these differences may explain our result that there was no correlation between the respondents’ replies to the two dilemma types. In fact, our analysis is very similar to Matisonn’s and concludes in the same way. The point of disagreement is whether there is any use in investigating this at all.

Trolley problems are generally seen as illustrating the complexities of doing versus allowing harm and how the conflict between deontological and consequentialist views may play out in various hypothetical scenarios. Our experiment was designed to evaluate the degree to which people’s intuitions in the various trolley problems would correlate with the respondents’ moral choice in a more specific context, where deontological and consequentialist reasoning were relevant. Our aim was to test the degree of transferability of deontological-versus-consequentialist orientation from the sterile trolley context to a more contextualized one. We agree that one should not expect a one-to-one correspondence between the subjects’ responses in the two contexts, but we found it reasonable to expect that those subjects who were more-than-averagely willing to act in the trolley dilemmas would also be more-than-averagely willing to accept the vaccination projects, overall. The main reason to expect some level of correlation was that trolley problems and vaccine scenarios share the key characteristic of sacrificing some for the many. When testing vaccines, a group of research participants are exposed to a risk, so that people in general may benefit from safe vaccines.

Although it could be argued that the dilemmas are too different for us to expect any correlation between people’s responses, it is not self-evident that the intuitions people have in trolley problems would not carry over, despite contextual noise. Other studies have shown that people’s intuitions in trolley problems do correspond to their moral judgments in monetary dilemmas and in scenarios that involve various types of harm (Bostyn et al., 2019; Dickinson & Masclet, 2018; Gold et al., 2013). It is of interest to investigate whether this would be the case also for more contextual medical ethics problems. It could have been the case that people’s consequentialist or deontological leanings mapped in trolley dilemmas were so strong that they at least would have some correlation to what people would think about the more complex dilemmas despite a richer context.

Thus, taking Matisonn’s points into account, we nevertheless find it worthwhile to investigate whether intuitions in the hypothetical trolley problem scenarios will carry over to moral decisions in more contextualized situations. Even if trolley problems were not designed with this use in mind, we should not exclude the possibility that intuitions uncovered by so-called “intuition pumps” shine through in more complex scenarios. At least, when we find out that this is not the case, it is not an uninteresting result.

An interesting discussion that Matisonn touches upon regards the role we should let intuitions play in philosophical discussions of morality. Relevant research in ethics and moral psychology questions the reliability of our intuitions, and discussions about methods and epistemology in philosophy indicate that ethics should not, and need not, rely on intuitions (Cappelen, 2012). This is, however, an on-going methodological debate in philosophy, and it does not seem irrelevant to present empirical data that provide some evidence supporting the current doubt, which we do in our articles.
